# A novel role for the tumour suppressor Nitrilase1 modulating the Wnt/β-catenin signalling pathway

**DOI:** 10.1038/celldisc.2015.39

**Published:** 2016-01-05

**Authors:** Sonnhild Mittag, Tomas Valenta, Jörg Weiske, Laura Bloch, Susanne Klingel, Dietmar Gradl, Franziska Wetzel, Yuan Chen, Iver Petersen, Konrad Basler, Otmar Huber

**Affiliations:** 1 Institute of Biochemistry, Department of Biochemistry II, Jena University Hospital, Friedrich-Schiller-University Jena, Jena, Germany; 2 Institute of Molecular Life Sciences, University of Zurich, Zurich, Switzerland; 3 Institute of Laboratory Medicine and Pathobiochemistry, Charité—Universitätsmedizin Berlin, Berlin, Germany; 4 Center for Sepsis Control and Care (CSCC), Jena University Hospital, Jena, Germany; 5 Department of Cell and Developmental Biology, Zoological Institute, Karlsruhe Institute of Technology, Karlsruhe, Germany; 6 Institute of Pathology, Jena University Hospital, Friedrich-Schiller-University Jena, Jena, Germany

**Keywords:** catenin, colorectal cancer, DNitFhit, nitrilase1, Rosetta-Stone hypothesis, tumour suppressor gene, Wnt signalling

## Abstract

Nitrilase1 was classified as a tumour suppressor in association with the fragile histidine-triad protein Fhit. However, knowledge about nitrilase1 and its tumour suppressor function is still limited. Whereas nitrilase1 and Fhit are discrete proteins in mammals, they are merged in *Drosophila melanogaster* and *Caenorhabditis elegans*. According to the Rosetta-Stone hypothesis, proteins encoded as fusion proteins in one organism and as separate proteins in another organism may act in the same signalling pathway. Although a direct interaction of human nitrilase1 and Fhit has not been shown, our previous finding that Fhit interacts with β-catenin and represses its transcriptional activity in the canonical Wnt pathway suggested that human nitrilase1 also modulates Wnt signalling. In fact, human nitrilase1 forms a complex with β-catenin and LEF-1/TCF-4, represses β-catenin-mediated transcription and shows an additive effect together with Fhit. Knockdown of human nitrilase1 enhances Wnt target gene expression. Moreover, our experiments show that β-catenin competes away human nitrilase1 from LEF-1/TCF and thereby contributes to the activation of Wnt-target gene transcription. Inhibitory activity of human nitrilase1 on vertebrate Wnt signalling was confirmed by repression of Wnt-induced double axis formation in *Xenopus* embryogenesis. In line with this finding, the *Drosophila* fusion protein *Drosophila* NitFhit directly binds to Armadillo and represses the Wingless pathway in reporter gene assays. Genetic experiments confirmed the repressive activity of *Drosophila* NitFhit on Wingless signalling in the *Drosophila* wing imaginal disc. In addition, colorectal tumour microarray analysis revealed a significantly reduced expression of human nitrilase1 in poorly differentiated tumours. Taken together, repression of the canonical Wnt pathway represents a new mechanism for the human nitrilase1 tumour suppressor function.

## Introduction

Human nitrilase1 (hNit1) was recognized during the characterization of the human fragile histidine triad (Fhit) tumour suppressor protein, when it was realized that in *D. melanogaster* and *C. elegans* Fhit is expressed as a C-terminal fusion protein with the unrelated protein nitrilase (Nit). Sequence alignments identified hNit1 as the closest homologue of the Nit domain in the *Drosophila* and *C. elegans* NitFhit fusion proteins exhibiting about 50% identity at the amino acid sequence level within the Nit domain [1]. The *C. elegans* NitFhit fusion protein forms a tetrameric complex with four Nit domains building the central core and the C-terminal Fhit domains aligning as dimers at opposing sites of the Nit core [2]. A similar dimeric structure was previously reported for the human Fhit protein [3]. Moreover, Fhit and Nit1 show similar expression patterns [4]. Based on these observations, Fhit and Nit1 were defined as Rosetta-Stone proteins [5] with a postulated common tumour suppressive function, although a direct interaction of both proteins has not been shown till now.

In contrast to Fhit, little is known about the function of Nit1 and interaction partners have not been studied. Together with nitrilase2 and the NitFhit fusion proteins of *D. melanogaster* and *C. elegans*, Nit1 is a member of the 10th out of 13 branches of the Nit protein superfamily. Only branch 1 enzymes are known to have specific nitrilase activity performing nitrile hydrolysis, whereas most other branches have apparent or postulated amidase or amide condensation activities [6]. Mammalian Nit1 was reported to cleave several synthetic dipeptide substrates, but has no amidase activity as reported for mammalian nitrilase2 [7, 8]. To date, no physiological substrate of Nit1 is known.

Nit1^−/−^ mice developed normally and revealed similar phenotypes as reported for Fhit^−/−^ mice such as accelerated cell proliferation, increased cyclin D1 expression and higher N-nitrosomethylbenzylamine-induced tumour incidence. Overexpression of Nit1 similar to Fhit overexpression resulted in enhanced apoptosis. Mutation of the Cys residue within the catalytic triad did not affect the tumour suppressive activity of Nit1 [4]. Moreover, it was shown that Nit1 acts as a negative regulator of T-cell proliferation [9]. Interestingly, overexpression of Fhit induces apoptosis in Nit1^−/−^ cells and vice versa, suggesting that both proteins can promote pro-apoptotic signalling independently [4]. In oesophageal adenocarcinoma, a loss of Nit1 expression was observed in 48% of the cases, suggesting a role in human cancer development [10]. However, loss of Nit1 and Fhit expression did not correlate in this study [10]. Currently, little is known about the mechanisms of Nit1 tumour suppressor function.

β-Catenin acts as a multifunctional adaptor protein, for example, within the cadherin–catenin cell–cell adhesion complex and in the canonical Wnt signalling pathway, thereby regulating diverse cellular processes including morphogenesis, proliferation, differentiation, tumourigenesis and neurodegenerative disease [11–14]. This requires that β-catenin level and function have to be tightly controlled. Excess cytoplasmic β-catenin is rapidly marked for degradation by the β-catenin destruction complex composed of the adenomatous polyposis coli (APC) protein, Axin, Dishevelled and the Ser/Thr kinases casein kinase 1 and glycogen synthase kinase-3-β. Activation of the Wnt pathway by binding of secreted members of the Wnt family to Frizzled/LRP6 receptor complexes in turn destabilizes the destruction complex and finally results in accumulation of β-catenin in the cytosol and its translocation into the nucleus. There, in association with LEF-1/TCF transcription factors β-catenin recruits further components including Bcl-9, Pygopus, Parafibromin and CREB-binding protein, which modulate transcription at promoters of target genes such as *c-myc*, *cyclin D1*, *sp5*, *axin2*, *survivin* and *MMP-14* [15].

Previously, we have identified β-catenin as a direct Fhit interaction partner [16, 17]. Based on this observation and in the context of a putative cooperation of Nit1 and Fhit as postulated by the Rosetta-Stone hypothesis [18], we here addressed whether hNit1/*Drosophila* NitFhit (dNitFhit) has a modulatory role in the canonical Wnt/Wingless (Wg) pathway by both biochemical and genetic analyses.

## Results

### Human Nit1 interacts with β-catenin/LEF-1 and represses Wnt signalling

**Figure 1:**
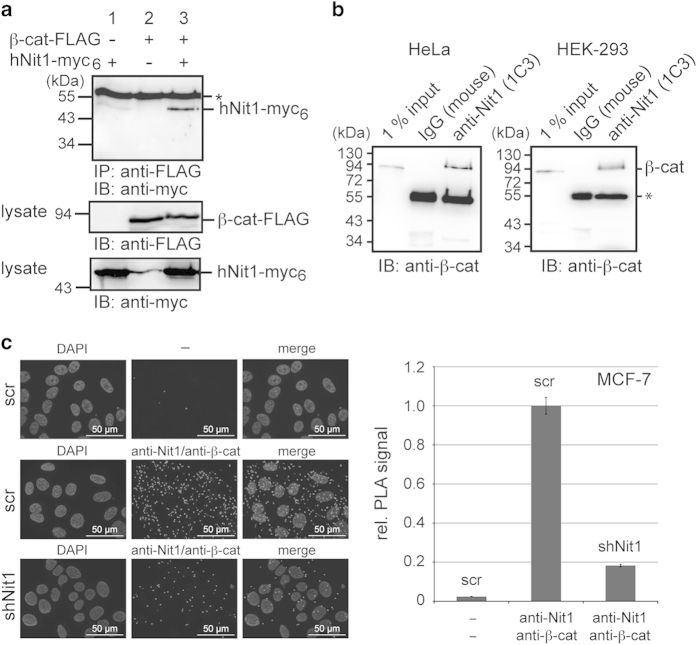
hNit1 interacts with β-catenin. (**a**) FLAG-tagged β-catenin forms a complex with myc_6_-tagged hNit1 in co-immunoprecipitation experiments with anti-FLAG M2 antibody. (**b**) Endogenous hNit1/β-catenin complexes can be co-immunoprecipitated from lysates of HeLa and HEK-293 cells with monoclonal anti-Nit1 (1C3) antibody. (**c**) PLAs using monoclonal anti-Nit1 (1C3) and polyclonal anti-β-catenin (M14M) antibodies confirm intracellular interaction of hNit1 with β-catenin. shNit1, MCF-7 clone 6 stably transfected with shNit1 construct; scr, MCF-7 cells stably transfected with a scrambled short hairpin RNA (shRNA) control construct; ‘—’, both primary antibodies omitted. All images are representatives of at least three independent experiments. Lysate controls are presented in the lower panels. *Heavy chain of precipitating antibody.

To test whether hNit1 can form a complex with β-catenin, co-immunoprecipitation experiments were performed in HEK-293 cells transiently transfected with β-catenin-FLAG and hNit1-myc_6_. As shown in Figure 1a, anti-FLAG-M2 antibody co-precipitated hNit1-myc_6_ in cells co-transfected with both constructs but not in controls that were transfected with only a single plasmid. Moreover, using the monoclonal anti-Nit1 (1C3) antibody it was possible to precipitate endogenous hNit1/β-catenin complexes from lysates of HeLa and HEK-293 cells (Figure 1b). Similar results were obtained with a polyclonal anti-Nit1 antibody in HEK-293 HeLa, HCT116 and MCF-7 cells (not shown). Proximity ligation assays (PLAs) [19] further confirmed an endogenous interaction of β-catenin and hNit1 within MCF-7 cells. Knockdown of endogenous hNit1 significantly reduced the PLA signals both in the cytoplasm and in the nucleus (Figure 1c). Immunofluorescence microscopy also showed cytosolic and nuclear localization of overexpressed hNit1 (Supplementary Figure S1). In nuclear/cytosolic fractionation experiments, overexpressed hNit1 predominantly localized in the cytosol with lower amounts localized in the nucleus. In these assays, overexpression did not change β-catenin cytosolic/nuclear distribution (Supplementary Figure S2A). Interestingly, when HEK-293 cells were stimulated with Wnt3a-conditioned medium, β-catenin in the nucleus increased and overexpression of hNit1 apparently decreased the amount of nuclear β-catenin (Supplementary Figure S2B). In addition, hNit1 was co-precipitated from lysates of HEK-293 cells transfected with FLAG-LEF-1 and hNit1, suggesting that hNit1 may associate with the β-catenin/LEF-1 transcription complex (Supplementary Figure S3).

**Figure 2:**
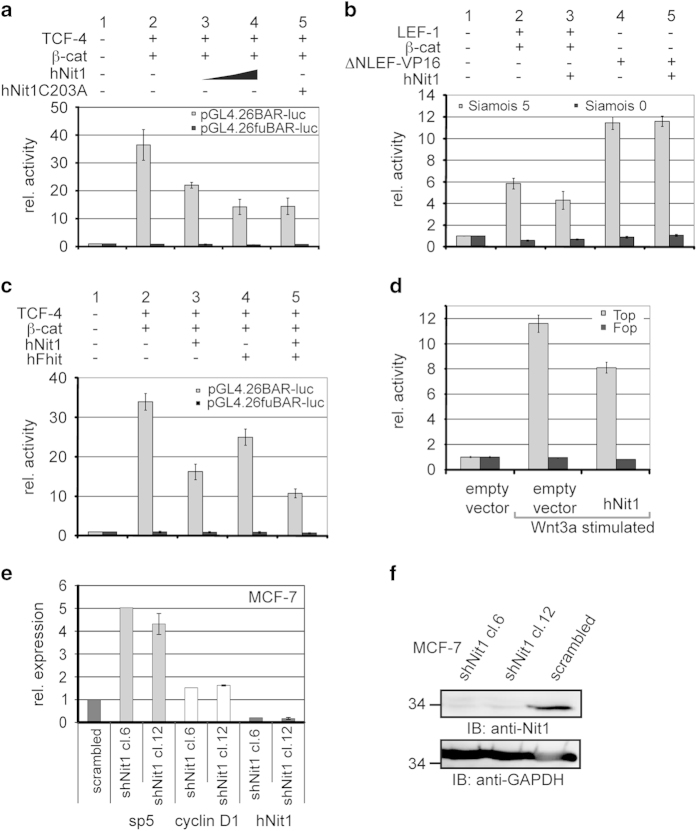
HNit1 and hFhit repress β-catenin-mediated transcription. HEK-293 cells were transiently transfected with the respective reporter gene constructs pGL4.26BARluc/pGL4.26fuBARluc or pGL3Siamois-luc S5/S0. BAR-luc/fuBAR-luc reporter gene expression was activated by transfection of TCF-4 and β-catenin expression plasmids. The phRL-Null *Renilla* luciferase plasmid was used to normalize transfection efficiency. Luciferase activities were measured 24 h after transfection. For all experiments, at least three independent transfections measured in duplicate and ±s.e.m. are presented. (**a**) Effects of hNit1 (0.5 μg or 1 μg) and enzymatic-dead hNit1C203A (1 μg) on β-catenin transcriptional activity. (**b**) Siamois S5/S0 reporter gene activity is reduced after co-transfection of hNit1 in LEF-1/β-catenin but not in ΔN-LEF-VP16-transfected cells. (**c**) Repression of BAR-luc activity measured after transfection of hNit1 and hFhit alone, and additive effect after co-transfection of both constructs. (**d**) Topflash/Fopflash reporter gene activity is repressed by hNit1 in Wnt3a-stimulated cells. (**e**) Expression of endogenous Wnt target genes *sp5* and *cyclin D1* is upregulated in hNit1 knockdown MCF-7 cells. Relative mRNA expression was analysed by quantitative reverse-transcriptase PCR. Two hNit1 knockdown clones were analysed (clone 12, *n*=4±s.e.m.; clone 6, *n*=1) and normalized to values obtained from the scrambled clone. (**f**) Cell lysates were analysed by western blotting. Glyceraldehydes 3-phosphate dehydrogenase (GAPDH) was used as a loading control.

In this context, we next analysed in reporter gene assays whether hNit1 has an effect on β-catenin-mediated transcriptional acitivity. Transfection of increasing amounts of hNit1 resulted in a dose-dependent inhibition of the pGL4.26BAR-luc [20] reporter gene activity in HEK-293 cells. Mutation of the Cys residue in the catalytic centre of the protein (hNit1C203A) did not impair the repressive activity (Figure 2a). A similar repressive activity of hNit1 was detectable in SW480 colon carcinoma cells in which the Wnt pathway is constitutively active due to a mutation in APC (Supplementary Figure S4). Comparable results were obtained when reporter gene assays were performed with Siamois-luciferase reporter gene constructs (S5 and S0) containing an endogenous promoter of a known β-catenin target gene [21] (Figure 2b, columns 1–3). Transcriptional activation induced by ΔNLEF-VP16, a construct that drives β-catenin-independent transcription via the herpes simplex virus VP16 transactivation domain [22], was not inhibited by hNit1 (Figure 2b, columns 4–5), indicating that hNit1 has not a general inhibitory effect on transcription but depends on β-catenin. As it has been reported that Nit1 and Fhit act additively [10], we next wanted to address whether an additive effect is also detectable in respect to β-catenin-mediated transcription. Indeed, co-transfection of both hNit1 and hFhit induced further repression (Figure 2c). Interestingly, expression of an artificial hNit1Fhit fusion construct, where the hNit1 protein was fused with hFhit by a linker peptide taken from the dNitFhit fusion protein, resulted in a similar repressive activity as detected for the co-expressed separate proteins (Supplementary Figure S5). Moreover, overexpression of hNit1 repressed reporter gene activation in response to Wnt3a stimulation (Figure 2d). By contrast, when hNit1 was knocked down by stable transfection of a short hairpin RNA construct in MCF-7 cells, expression of the endogenous Wnt target genes *sp5*, *cyclin D1* (Figure 2e) and *MMP-14* (Supplementary Figure S6) was increased. Efficiency of knockdown was analysed by quantitative reverse-transcriptase PCR (Figure 2e) and western blotting (Figure 2f) in two different clones. To examine whether hNit1 is recruited to promoters of TCF/β-catenin target genes, chromatin immunoprecipitation experiments were performed, showing that endogenous hNit1 indeed is associated with the *cyclin D1* and *MMP-14* promoters (Supplementary Figure S7). From these data we conclude that both hNit1 and hFhit repress β-catenin-mediated transcription in an additive manner.

### HNit1 represses double axis formation in *Xenopus*

**Figure 3:**
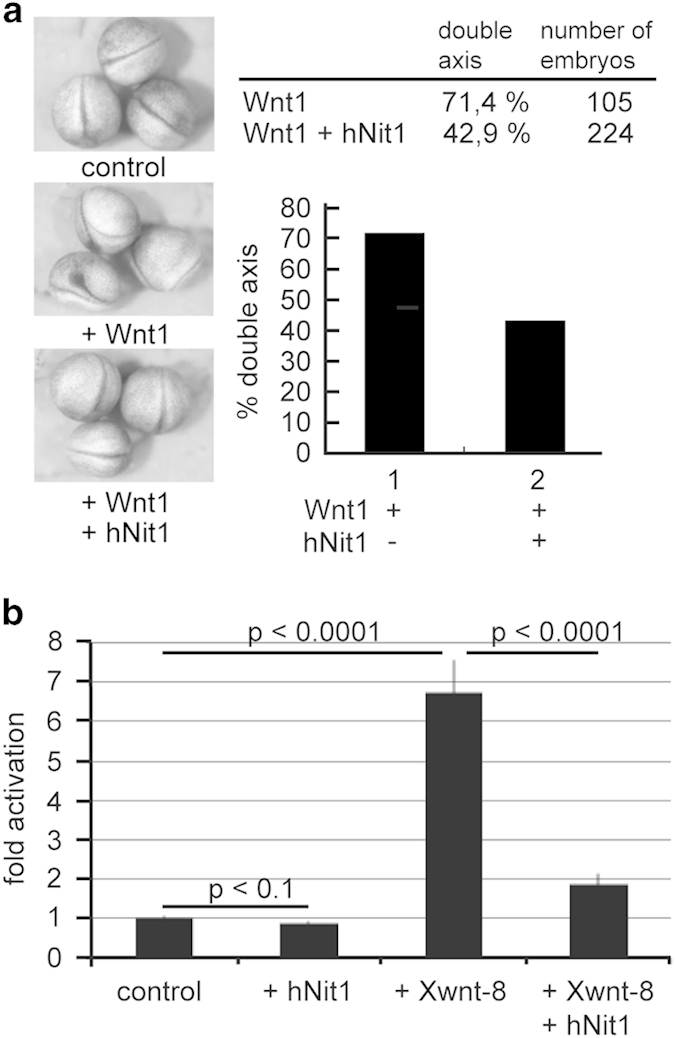
HNit1 represses Wnt1-induced double axis formation in *X. laevis* embryos. (**a**) Injection of 5 pg mWnt1 mRNA into the marginal zone of *Xenopus* four-cell stage embryos induced in 71.4% of the injected embryos an ectopic axis. This is best seen by the Y-shaped duplication of the neural tube. Co-injection of 500 pg hNit1 reduced the frequency of secondary axis formation to 42.9%. Examples of embryos showing inhibited secondary axis (+ Wnt1) formation when hNit1 RNA is injected. (**b**) *Xenopus* embryos were injected with a Siamois-luciferase reporter plasmid and the indicated combinations of hNit1 and XWnt8 mRNAs. *Siamois* promoter-dependent transcription was reduced when hNit1 mRNA was co-injected.

Next, we addressed whether Nit1 also acts as a repressor of the canonical Wnt signalling pathway in vertebrates *in vivo* by analysing secondary axis formation in *Xenopus laevis*, a standard model to investigate Wnt/β-catenin signalling. Injection of Wnt1 mRNA typically induces ectopic axis formation in *Xenopus* embryos. Co-injection of hNit1 mRNA strongly abrogated the frequency of secondary axes (Figure 3a). In contrast, co-injection of prolaktin mRNA as a control had no effect (not shown). To further confirm this observation, a Siamois-luciferase reporter construct was injected and activation of this construct in response to co-injection of XWnt8+/−hNit1 mRNAs was quantified. Consistent with the reporter gene assays in transfected HEK-293 cells, hNit1 acts as a repressor of Siamois promoter-dependent transcription (Figure 3b). These results show that hNit1 also acts as a repressor of canonical Wnt signalling *in vivo*.

### HNit1 directly binds to the Armadillo repeat region in β-catenin

**Figure 4:**
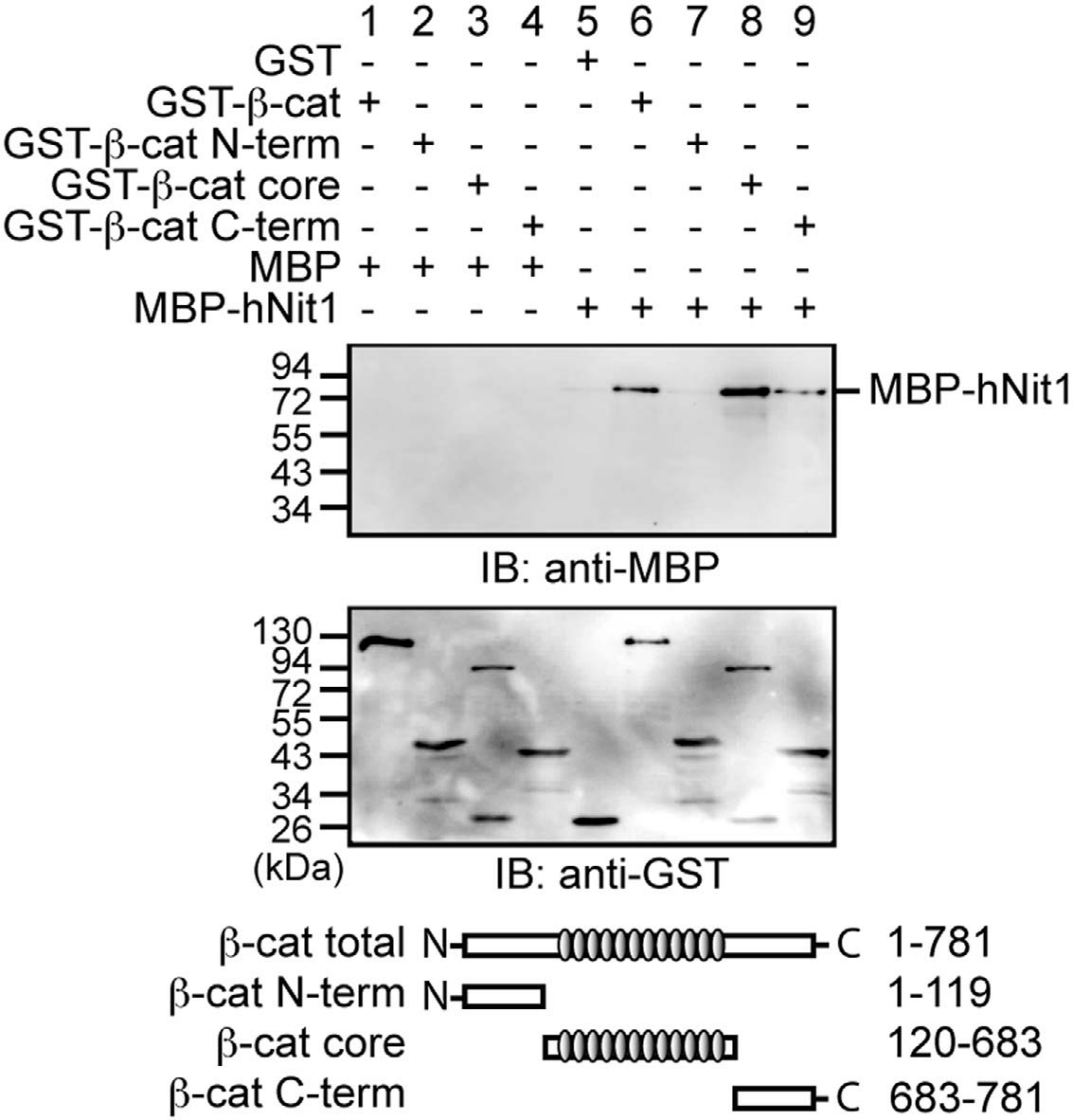
HNit1 directly binds to the Armadillo-repeat region in β-catenin. *In vitro* pull-down assay were performed with purified recombinant GST-β-catenin and MBP-hNit1 fusion proteins as indicated. GST and MBP were used as a control. Deletion constructs of β-catenin (GST-β-cat (aa1–781); GST-β-cat-N-term (aa1–119); GST-β-cat-core (aa120–683) and GST-β-cat-C-term (aa683–781)) were used to map the hNit1-binding site in β-catenin. Protein complexes were isolated with glutathione (GSH)-agarose beads and analysed by western blotting with a monoclonal anti-MBP or a polyclonal anti-GST antibody. The image is a representative of at least three independent experiments.

To investigate whether the observed repressive effect of hNit1 is mediated by a direct interaction with β-catenin, we performed *in vitro* association assays. A purified recombinant glutathione *S*-transferase (GST)-β-catenin fusion protein pulled down MBP (maltose-binding protein)-hNit1 (Figure 4, lane 6), revealing that the interaction between β-catenin and hNit1 is direct. MBP-hNit1 did not bind to a GST-ECT (E-cadherin cytoplasmic tail) fusion protein as a negative control (not shown). Using N- and C-terminal deletion constructs of β-catenin, the binding site for hNit1 was mapped to the Armadillo-repeat region of β-catenin. Minor binding of MBP-hNit1 was detectable to the β-catenin C-terminal domain (Figure 4).

**Figure 5:**
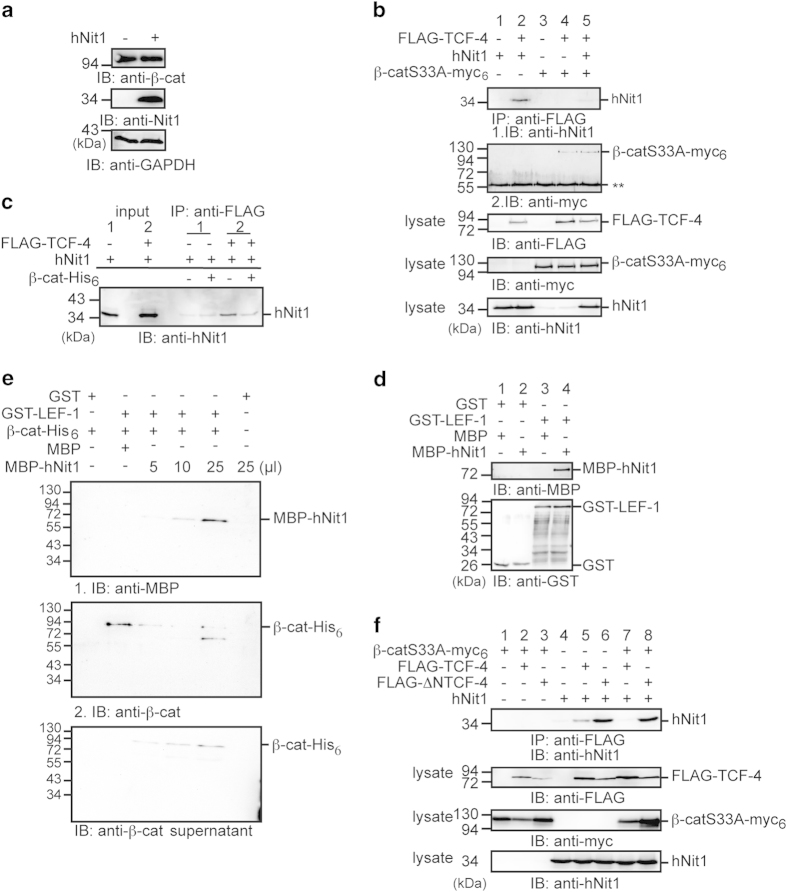
HNit1 binds to LEF-1/TCF-4 and competed with β-catenin. (**a**) Transient overexpression of hNit1 does not affect total β-catenin levels in HEK-293 cells. Glyceraldehydes 3-phosphate dehydrogenase (GAPDH) was used as a loading control. (**b**) Co-transfection of β-catenin removes hNit1 from TCF-4. HEK-293 cells were transiently transfected with β-catenin-S33A-myc_6_, FLAG-TCF-4 and hNit1 as indicated. Co-immunoprecipitations were performed with anti-FLAG M2 antibody and precipitated protein complexes were analysed by western blotting with anti-hNit1 or anti-myc (9E10) antibodies. Lysate controls are shown in the lower panels. **Heavy chain. (**c**) β-Catenin-His_6_ dissociates hNit1/TCF-4 complexes in cell lysates. HEK-293 cells were transfected with FLAG-TCF-4 and/or hNit1 and lysed 48 h after transfection. Purified recombinant β-catenin-His_6_ was added to the lysate as indicated and subsequently immunprecipitation with the anti-FLAG M2 antibody was performed. Isolated protein complexes were analysed by western blotting with the anti-hNit1 antibody. (**d**) HNit1 interacts directly with LEF-1. Binding of GST-LEF-1 to MBP-hNit1 was analysed in pull-down assays. Protein complexes were isolated with glutathione (GSH)-agarose beads and subsequently analysed by western blotting with an anti-MBP antibody. GST and MBP were used as a control. (**e**) MBP-hNit1 dissociates preformed purified GST-LEF-1/β-catenin-His_6_ complexes. Concomitant with increasing amounts of MBP-Nit1 more MBP-Nit1 is binding to GST-LEF-1 and β-catenin-His_6_ is released into the supernatant (lower blot). The middle blot is identical to the blot in the upper image, which after first treatment with anti-MBP antibody was washed and subsequently treated with anti-β-catenin antibody. (**f**) HNit1 associated with FLAG-ΔNTCF-4 is not dissociated by β-catenin. Each experiment was performed at least three times.

In a next step, we aimed to examine the mechanism by which hNit1 represses β-catenin signalling. Overexpression of hNit1 did not change the β-catenin protein levels within cells, indicating that hNit1 did not affect β-catenin stability (Figure 5a). Binding of hNit1 to the Arm-repeat domain in β-catenin suggested that hNit1 impairs formation of TCF/LEF-1/β-catenin transcription complexes required for target gene activation. To test this, HEK-293 cells were transfected with different combinations of a constitutive active mutant of β-catenin (β-cat-S33A-myc_6_) and FLAG-TCF-4 together with hNit1. After immunoprecipitation with anti-FLAG-M2 antibody, hNit1 associates with TCF-4 in the absence of co-transfected β-catenin-S33A-myc_6_ (Figure 5b, lane 2) in line with the finding shown in Supplementary Figure S1A that hNit1 forms a complex with LEF-1. However, when β-catenin-S33A-myc_6_ was co-transfected, only minor amounts of hNit1 co-precipitated with FLAG-TCF-4 (Figure 5b, lane 5). Similar results were obtained, when lysates of HEK-293 cells co-transfected with FLAG-TCF-4 and hNit1 were incubated with purified recombinant β-catenin-His_6_ before immunoprecipitation. Addition of β-catenin-His_6_ significantly reduced association of hNit1 with TCF-4 (Figure 5c). These observations suggested that hNit can directly bind to TCF/LEF transcription factors, and that β-catenin and hNit1 compete for binding to TCF-4. Pull-down assays with purified recombinant GST-LEF-1 and MBP-Nit1 revealed a direct interaction of LEF-1 and Nit1 (Figure 5d). Moreover, competition for LEF-1 was confirmed in pull-down assays with purified recombinant GST-LEF-1, β-catenin-His_6_ and MBP-hNit1 proteins. A GST-LEF-1/β-catenin-His_6_ complex was preformed and isolated by pulldown of GST-LEF-1 with glutathione-agarose beads. After washing, complexes were incubated with increasing amounts of MBP-hNit1 and washed again. Addition of MBP-hNit1 resulted in increased binding of MBP-hNit1 to GST-LEF-1 and a concomitant release of β-catenin-His_6_ to the supernatant (Figure 5e).

Concerning the observed competition, we next addressed whether hNit1 binds to the N-terminal β-catenin-binding domain in LEF-1/TCF [23–25]. Interestingly, in co-immunoprecipitation experiments from lysates of HEK-293 cells transiently transfected with either FLAG-TCF-4 or an N-terminal-deleted FLAG-ΔNTCF-4 construct, hNit1 showed stronger binding to the N-terminal-deleted TCF-4 construct than to the full-length TCF-4 (Figure 5f, lanes 5 and 6). Moreover, co-expression of β-catenin-S33A-myc_6_ impaired binding of hNit1 to full-length TCF-4 as shown above, whereas β-catenin-S33A-myc_6_ was not able to disrupt the interaction of hNit1 with ΔNTCF-4 (Figure 5f, lanes 7 and 8). Taken together, these data suggest that hNit1 in association with LEF-1/TCF transcription factors can act as a co-repressor of canonical Wnt signalling and increasing concentrations of β-catenin titrate away hNit1 from LEF-1/TCF transcription factors.

### The dNitFhit protein interacts with and represses Armadillo transcriptional activity

**Figure 6:**
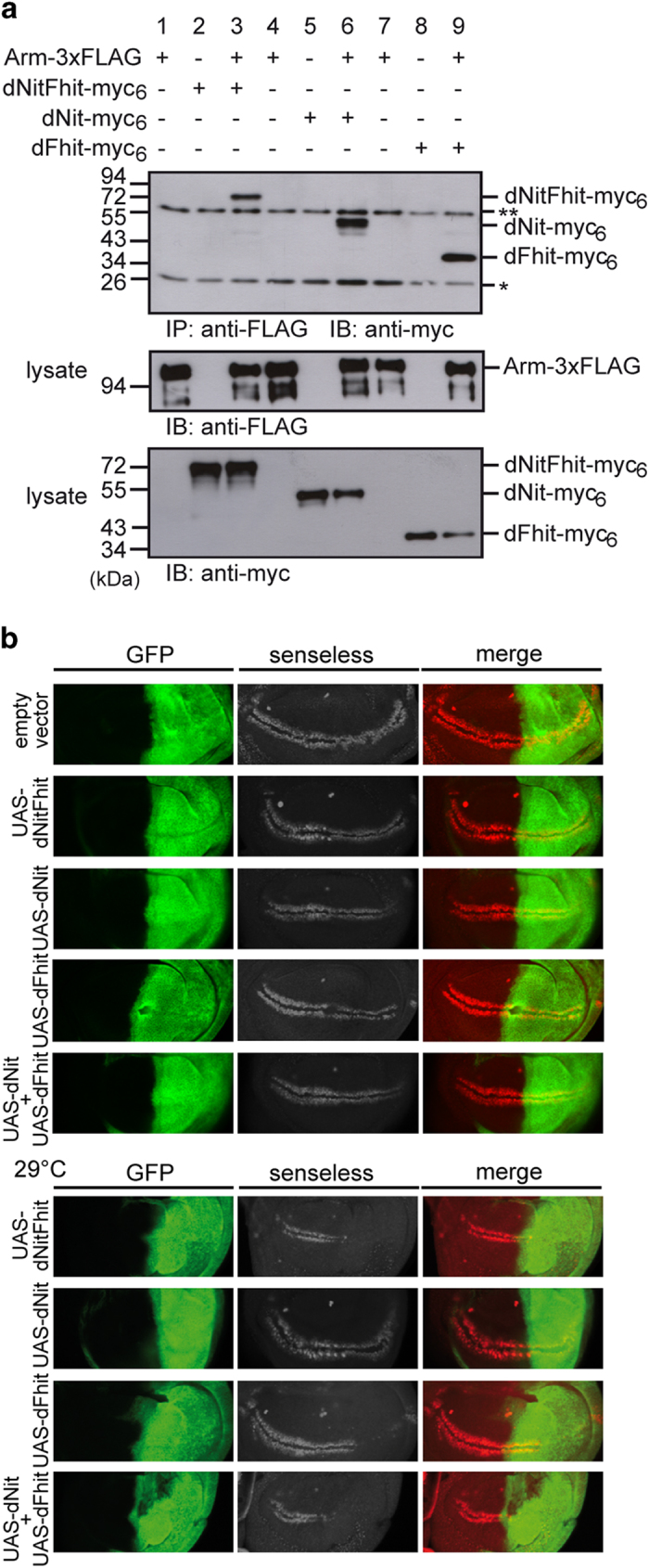
The Rosetta-Stone fusion protein dNitFhit binds to Armadillo and β-catenin, and represses Wg-mediated transcription. (**a**) FLAG_3_-Armadillo and dNitFhit-myc_6_ form a complex in co-immunoprecipitations from lysates of transiently transfected HEK-293 cells. Similar complex formation was observed for the dNit and dFhit domain expressed alone. Protein complexes were analysed by western blotting with anti-myc (9E10) antibody. Lysate controls are shown in the lower panels. *Light chains, **heavy chains of the precipitating antibody. (**b**) *Engrailed-Gal4*-driven expression of *UAS-dNitFhit*, *UAS-dNit*, *UAS-dFhit* or combined *UAS-dNit+UAS-dFhit* repress *Senseless* expression in *Drosophila* wing imaginal discs. The repressive effect is manifested as reduced intensity and width/thickness of immunostained Senseless pattern. Coexpression of *UAS-GFP* was used to mark the posterior part of the wing discs where the *Engrailed-Gal4* driver is active. Transgenic fly line expressing empty vector was used as a wild-type control. Analysis of *Senseless* expression at 29 °C is enhancing the effect as shown in the lower panels.

To further confirm these observations and to decipher whether inhibition of Wnt/β-catenin signalling by NitFhit is an evolutionary conserved mechanism, we investigated whether the dNitFhit fusion protein affects the Wg signalling pathway in a similar manner. In this respect, we analysed whether dNitFhit associates with the *Drosophila* β-catenin homologue Armadillo in co-immunoprecipitation experiments with lysates obtained from HEK-293 cells transiently transfected with dNitFhit-myc_6_ and Armadillo-3xFLAG alone or in combination. DNitFhit/Armadillo complexes were readily detectable in cells transfected with both constructs but not when transfected with single construct as a control (Figure 6a, lanes 1–3). In a next step, we wanted to examine which domain of dNitFhit mediates binding to Armadillo. To this end, we transfected HEK-293 cells with FLAG_3_-tagged Armadillo and either the myc_6_-tagged dNit or dFhit domain of dNitFhit. In immunoprecipitations with an anti-FLAG antibody, both the dFhit and the dNit domain were co-precipitated (Figure 6a, lanes 4–9). Moreover, dNitFhit also formed a complex with β-catenin in co-immunoprecipitation experiments (Supplementary Figure S8A), suggesting that this interaction is highly conserved in evolution. To prove that this interaction is direct, pull-down assays with purified recombinant fusion proteins were performed. In these experiments, MBP-dNitFhit specifically associated with GST-Armadillo and GST-β-catenin but not with GST alone (Supplementary Figure S8B and C). Taken together, these findings suggested that dNitFhit in forming a complex with Armadillo may modulate Wg signalling in *Drosophila* as shown for hNit1 above.

The functional relevance of the interaction between dNitFhit and Armadillo was tested in more detail using the Wg/Armadillo transcriptional reporter *wingful*-luciferase in *Drosophila* Kc− or S2R+ cells. Both full-length dNitFhit and its sub-parts dFhit and dNit alone significantly repressed reporter gene activity in a dose-dependent manner. Interestingly, the seperated dNit and dFhit domains alone had a weaker repressive effect, but co-expression of both showed an additive effect (Supplementary Figure S8D).

### DNitFhit acts as a repressor of Wg signalling *in vivo*

As a next step we studied this repressive role *in vivo*. Using the attP/ΦC31 integration system we generated transgenic *Drosophila* lines expressing dNitFhit (and dNit or dFhit) under the control of the heterologous UAS promoter. To assess the repressive activity of UAS-dNitFhit we focused on the expression of the high threshold Wg/Armadillo target gene *senseless*. In the wild-type wing, imaginal disc of third larval instar senseless is expressed in two well-defined stripes along the dorsoventral boundary in close proximity to the Wg-secreting cells [26]. The overexpression of UAS-dNitFhit driven by *engrailed-Gal4* driver in the posterior part of the wing discs dramatically reduced Senseless expression. The levels of senseless are decreased in the part where dNitFhit is overexpressed, in comparison with the anterior part that remains as wild type. This repressive effect was also apparent when the separated domains dNit and dFhit were overexpressed alone, but appeared more pronounced when Nit and Fhit were overexpressed together, either as dNitFhit fusion protein or co-expressed (Figure 6b, upper panel). As the heterologous Gal4/UAS system is temperature sensitive, we also tested the effects at higher temperatures. Indeed, if NitFhit or Nit and Fhit were expressed via *engrailed-Gal4* at 29 °C, the expression of Wg target senseless in the posterior compartment was almost completely blocked (Figure 6b, lower panel). Taken together, these genetic experiments clearly confirm our hypothesis that dNitFhit can act as a repressor of Wg signalling, and that this repression is a conserved mechanism to balance Wnt/β-catenin-regulated target gene activation.

### HNit1 expression is reduced in colorectal cancer

**Figure 7:**
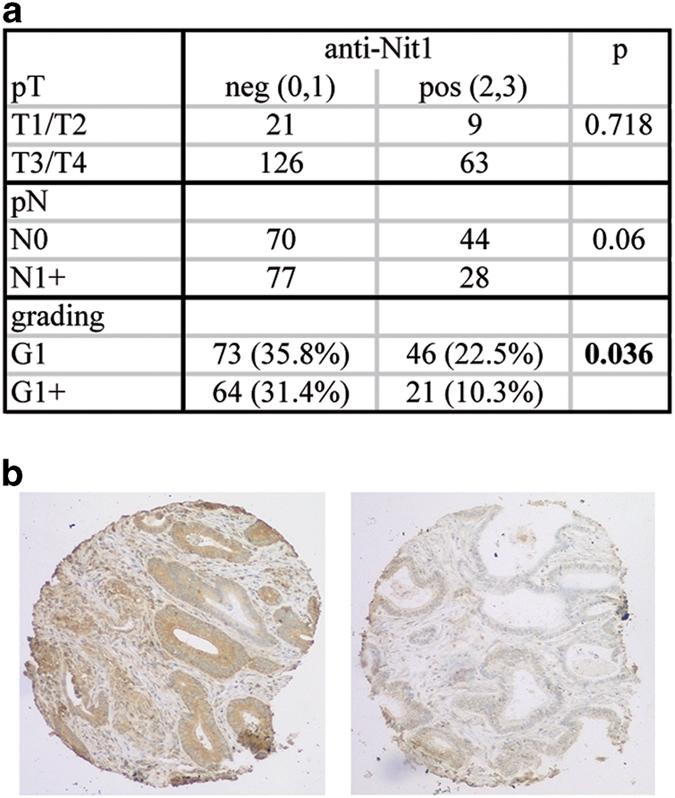
HNit1 expression is reduced in dedifferentiated human colon cancer. Tissue microarrays with 219 human colorectal carcinoma samples were stained for hNit1 using the monoclonal anti-nitrilase1 (1C3) antibody. (**a**) Frequencies of negative (0), weakly positive (1), moderately positive (2) and strongly positive (3) stainings in tumour samples according to tumour staging. (**b**) Immunohistochemical staining of hNit1 in a well- (left) and a poorly differentiated (right) colorectal carcinoma (×100 magnification).

In colon cancer, the canonical Wnt pathway is aberrantly activated at high frequency [27]. Thus, deregulated expression of hNit1 may further enhance tumourigenesis. Therefore, we investigated whether Nit1 expression is altered in human cancer. Immunohistochemical analyses of 219 colorectal carcinoma samples on tissue microarrays detected hNit1 staining predominantly in the cytoplasm and to a low amount also in the nucleus (Figure 7). *χ* ^2^-test using software package SPSS 13.0 (SPSS, Chicago, IL, USA) revealed that in well-differentiated tumours hNit1 staining was significantly more intense compared with poorly differentiated tumours (*P*=0.036), consistent with its tumour suppressor function. Although a high number of pN1+ tumours showed reduced staining of hNit1, correlation with involvement of lymph nodes or patient survival was not statistically significant (*P*=0.06 and *P*=0.298, respectively).

## Discussion

We have previously characterized the fragile histidine-triad protein hFhit as a new β-catenin interaction partner and repressor of β-catenin transcriptional activity [16, 17], providing a new molecular mechanism for the hFhit tumour suppressor function. In lung cancer, a strong correlation exists for loss of hFhit expression and overall survival, both in adenocarcinoma and squamous cell cancers. In addition, cytoplasmic accumulation of β-catenin in adenocarcinoma and MUC1 expression in squamous cell cancer was associated with shorter survival [28]. In the context of our studies, this implies that loss of Fhit expression contributes to enhanced β-catenin signalling and worse prognosis in lung cancer. Interestingly, in *Drosphila* Fhit is expressed as a fusion protein with Nit1 defining dNitFhit as a Rosetta-Stone protein [5]. The Rosetta-Stone hypothesis predicts that proteins, expressed as a fusion protein in one organism, are functionally linked in the organisms where they are expressed as individual proteins. Having this in mind, we hypothesized that hNit1 may also modulate Wnt/β-catenin signalling. Here we show that hNit1 indeed interacts with β-catenin and negatively regulates β-catenin target gene expression in reporter gene assays and quantitative reverse-transcriptase PCR analysis of endogenous target genes. In line with the observed repressive activity, hNit1 also inhibits Wnt-induced double-axis formation in *Xenopus* embryos. Moreover, co-transfection of hFhit augmented the hNit1 repressive effect in reporter gene assays, suggesting that both proteins are able to cooperate in signalling pathways such as the canonical Wnt pathway. In line with this, an artificial hNit1–hFhit fusion protein exhibits similar repressive activity as co-transfected hNit1 and hFhit. Such a combined and additive repressive effect also was observed in Nit1^−/−^Fhit^−/−^ double knockout mice [10].

In addition, the *Drosophila* dNitFhit Rosetta-Stone protein, where Fhit is C-terminally fused to the Nit part, similarly binds to the β-catenin homologue Armadillo and acts as a repressor of the Wg/Armadillo pathway. Interestingly, both subparts—dNit and dFhit—are able to independently repress Wg signalling and revealed an additive effect in *Drosophila* imaginal disc development, again supporting a common function as suggested by the Rosetta-Stone hypothesis. Furthermore, the analysis of the *Drosophila* fusion protein dNitFhit indicates that the Wnt/Wg-repressing activity of Nit and Fhit is evolutionary conserved.

In binding directly to the Arm-repeat region of β-catenin, hNit1 uses a different site compared with hFhit [17]. Therefore, it is possible that a ternary complex composed of hNit1, β-catenin and hFhit is formed. In contrast to hFhit, hNit1 in addition to β-catenin also can bind directly to LEF-1/TCF transcription factors. Interestingly, our data suggest that hNit1 binds to a site that does not correspond to the β-catenin-binding site. Deletion of the N-terminal β-catenin binding site did not impair binding of hNit1 to TCF-4. The co-repressors TLE (transducin-like enhancer of split)/Groucho also bind to two sites different from the β-catenin-binding site [29] and are dissociated from LEF-1/TCF in response to Wnt stimulation. TLE acts as a tetramer on LEF/TCFs not by direct competition but by different affinities mediating an activation–repression switch [30]. Similarly, co-immunoprecipitation experiments showed that hNit1 dissociates from TCF-4 in response to overexpression of β-catenin. Coincidentally, hNit1 also acts as a tetramer, suggesting that similar to TLEs it may have a recruiting function, for example, of histone deacetylases (HDACs) [29]. Despite binding to both β-catenin and LEF-1/TCF, the repressive effect of hNit1 depends on β-catenin, as hNit1 was not able to repress reporter gene activity in response to ΔNLEF-VP16 [22], a β-catenin-independent constitutive active form of LEF-1. Moreover, this assay suggests that hNit1 does not impair binding of LEF-1/TCF to DNA. Although hNit1 is binding to the ΔNTCF-4 construct and thus interacts with a site different from the β-catenin-binding site, dissociation of hNit1 from LEF-1/TCF can be induced by β-catenin, as it was similarly shown for Groucho/TLE, which also uses an interaction site in LEF-1/TCF apart from the β-catenin-binding site [29]. Moreover, there is evidence that in addition to Groucho/TLE there are further LEF-1/TCF-associated co-repressors that regulate LEF-1/TCF-driven transcription [31]. Altogether, these observations suggest that hNit1 acts as a LEF-1/TCF-associated co-repressor, which is dissociated from LEF-1/TCF by increasing levels of β-catenin as similarly observed for p15RS [32]. Future studies are required to investigate the role of the Nit1 interaction with LEF-1/TCF in more detail. Different ways how hNit1 represses β-catenin signalling function can be considered. For example, hNit1 such as p15RS may be involved in the recruitment of specific HDACs [33]. HDACs were reported to form a complex with LEF-1/TCF transcription factors and β-catenin [34, 35], to mediate their repressive function. Moreover, HDAC1 was reported to be recruited to transcription regulatory complexes by Reptin [36, 37]. Otherwise, hNit1 can interfere with the binding of histone acetyltransferases p300 [38] and CREB-binding protein [39] or other activators such as Pontin [40, 41] or Bcl-9/Legless [42, 43] and parafibromin [44] to β-catenin. As another possibility, hNit1 may be involved in the recruitment of kinases such as casein kinase 1 [45] or Nemo-like kinase [46] that disrupt the β-catenin/TCF complex. In this context it is currently unknown whether and how hNit1 itself is regulated by posttranslational modifications.

The association of hNit1 with the *cyclin D1* promoter is in line with a previous report showing that Wnt target genes such as *cyclin D1* [47] or *claudin-1* [48] are upregulated in cells isolated from Nit1^−/−^ mice [4]. Consistently, we could show that a knockdown of hNit1 in MCF-7 cells resulted in increased mRNA levels of the Wnt target genes *cyclin D1*, *sp5* and *MMP-14* [47, 49, 50]. Thus, hNit1-dependent regulation of *cyclin D1* expression at least in part appears to be mediated by β-catenin and probably is also involved in the regulation of cell survival. However, the amount of hNit1 within the nucleus appears to be limited and tightly regulated. The mechanisms of this regulation remain to be investigated.

Analysis of colon carcinoma tissue microarrays revealed that hNit1 expression is significantly reduced in dedifferentiated tumour samples. Future studies are needed to unravel whether hNit1 mutations or loss of hNit1 expression occur in tumours at similar frequencies as reported for hFhit and how mutations of both hFhit and hNit1 affect outcome compared with mutations of a single gene. It will be interesting to see whether and how hNit1 expression is correlated with the β-catenin, APC and Ras status of tumours. Moreover, it currently is not clear how hNit1 expression itself is regulated.

In conclusion, our studies identified hNit1 and the dNitFhit fusion protein as conserved negative regulators of the canonical Wnt/Wg signalling pathway. As postulated by the Rosetta-Stone hypothesis, hNit1 and hFhit share β-catenin as a common interaction partner and appear to have an additive repressive effect on β-catenin transcriptional activity. Our data further confirm the postulated tumour suppressor function of hNit1 and provide a molecular mechanism for this activity.

## Materials and Methods

### Cell culture and antibodies

HEK-293, HeLa and MCF-7 cells were grown as reported previously [17]. Monoclonal anti-FLAG M2, anti-MBP (clone MBP-17) and anti-nitrilase1 (clone1C3) antibodies were purchased from Sigma (Schnelldorf, Germany), anti-β-catenin (clone 14) from BD Biosciences (Heidelberg, Germany), anti-M14M [51] and anti-glyceraldehyde-3-phosphate dehydrogenase (GAPDH) from Merck Millipore (Darmstadt, Germany) and anti-Fhit from Upstate Biotechnology (Millipore Corp., Billerica, MA, USA). A polyclonal rabbit anti-nitrilase1 antibody was generated at Pineda-Antikörper-Service Berlin by immunization with the nitrilase1-specific peptide CFHERGQDWEQTQKI coupled to KLH. Antibodies were affinity-purified on MBP-hNit1 coupled to Mini-Leak beads (KemEnTec, Taastrup, Denmark). Horseradish peroxidase-labelled secondary antibodies were purchased from Dianova (Hamburg, Germany). Colorectal cancer tissue microarray construction and immunohistochemistry were performed at the Institute of Pathology, Jena University Hospital, as described previously [52]. The study was approved by the ethical committee of the Friedrich-Schiller-University Jena (3815-07/13).

### Plasmids

Oligonucleotides used for PCR are summarized in Supplementary Table S1. Sequences of all constructs were confirmed by resequencing. HNit1 isoform 4 (NM_001185094) was used for all experiments. The indicated plasmids were generated using standard procedures. The endogenous *Bam*HI site and the Nitrilase1C203A mutations were performed with the QuikChange site-directed mutagenesis kit (Stratagene, Heidelberg, Germany). A human NitFhit fusion protein was generated by overlap extension PCR introducing the *Drosophila* linker sequence between hNit1 and hFhit. PCR products were ligated into pCS2+, pCS2+-myc_6_, pFLAG-CMV4, pGEX-4T1, pQlinkG [53] and pMAL-c2. DNitFhit cDNA was amplified from the intronless genomic DNA by PCR. The PCR products were ligated into the *Bam*HI sites of pMal-C2X, pCS2+myc_6_, p3xFLAG-CMV10, pUAST-attB and pAc5.1-FLAG. Plasmids encoding Fhit [17], GST-Armadillo [54], GST-β-catenin deletion constructs [51] and GST-LEF-1 [24] were described earlier. pCMV4-FLAG-TCF-4 and pCMV4-FLAG-ΔNTCF-4 were generated by PCR using pcDNAI hTCF-4 as a template. pGL4.26BAR-luc was generated by transferring the 12 LEF-1/TCF response elements in pBAR [20] into pGL4.26 (Promega, Heidelberg, Germany) using *Kpn*I/*Hind*III.

### Transient transfections, reporter gene and quantitative reverse-transcriptase PCR assays

Reporter gene assays were performed as described previously [17, 55]. HEK-293 cells grown in 6- (5×10^5^ cells per well) or 24- (1×10^5^ cells per well) well plates were transiently transfected with the indicated constructs. pGL4.26-BAR, -fuBAR, Siamois-luciferase (S5 and S0) [21] or Topflash/Fopflash (pGL3-OT/OF) plasmids were used as reporter constructs. Luciferase activity was measured 24 h after transfection with a dual luciferase reporter assay system. Wnt3a-conditioned medium was added 8 h after transfection and luciferase activities were measured 16 h later. Wnt3a-conditioned and control conditioned media were generated by Wnt3a-transfected and empty vector-transfected L-M(TK^−^) cells. Transfection efficiency was normalized by co-transfection of constitutive active *Renilla* luciferase (phRL-Null). Average values of at least three independent transfection experiments are presented for all reporter gene assays. *Drosophila* Kc (or S2R+) cells were transfected in 96-well plates by CellFectin (Invitrogen, Basel, Switzerland) with wf-Luciferase reporter, tubulin α1-*Renilla* and empty vector or dNitFhit constructs. Cells were stimulated with Wg-conditioned or control medium 48 h after transfection for 24 h and luciferase activities were measured in triplicate and normalized to *Renilla*-luc activity.

For hNit1 knockdown studies, MCF-7 cells were stably transfected with pGFP-V-RS short hairpin RNA Nit1 (GI344668) or pGFP-V-RS scrambled non-effective short hairpin RNA (TR30013) (OriGene Technologies, Inc., Rockville, MD, USA). Total RNA was isolated by using the NucleoSpin RNA II kit (Macherey & Nagel, Düren, Germany) and reverse transcription was performed with the High-Capacity cDNA Reverse Transcription Kit (Life Technologies, Darmstadt, Germany). Real-time PCR was done with UPL probes (Roche Applied Science, Mannheim, Germany) using the primer/probe combinations given in Supplementary Table S2. Each PCR was set up in duplicates and performed at least from three independent RNA preparations. Threshold cycle (*C*_t_) values of the target genes were normalized to the endogenous control glyceraldehyde-3-phosphate dehydrogenase. Differential expression was calculated according to the 2^−ΔΔ*C*^_t_ method.

### Immunoprecipitation and western blot analyses

For immunoprecipitation, 8×10^5^ HEK-293 cells per six well were transiently transfected with the indicated constructs in different combinations. After 48 h, cells were lysed with ice-cold lysis buffer (20 mM imidazole pH 8.0, 150 mM NaCl, 2 mM MgCl_2_, 300 mM sucrose, 0.25% (v/v) Triton X-100 and Complete protease inhibitor mix (Roche)) for 20 min at 4 °C and centrifugated (10 min, 20 800×*g*). Immunoprecipitation was performed with precleared lysate (200 μg of total protein) and 2 μg of the appropriate antibody pre-bound to Protein A Sepharose (GE Healthcare, Freiburg, Germany) as described [17]. To detect endogenous protein complexes, cells were lysed in ice-cold lysis buffer for 20 min at 4 °C. Subsequently, lysates were incubated for 10 min in an ultrasonic bath and centrifuged as above. For immunoprecipitation, 4 μg anti-nitrilase1 (clone1C3) or 3 μg of the polyclonal anti-Nit1 or antibody was incubated with cell lysate (500 μg of total protein) for 2 h under constant agitation at 4 °C and subsequently precipitated by addition of Protein A Sepharose. Immunoprecipitation and western blot analyses were performed as described [17] using the indicated antibodies (1 μg ml^−1^ anti-FLAG M2, 1:1 000 anti-myc (9E10), 0.25 μg ml^−1^ anti-Fhit and 0.1 μg ml^−1^ anti-β-catenin).

### Expression of GST and MBP fusion proteins and pull-down assays

All fusion proteins were expressed in *Escherichia coli* XL1 blue or BL21-RE4 grown in Luria broth media and induced with 1 mM isopropyl β-D-1-thiogalactopyranoside. For expression of GST-Armadillo, bacteria were induced in M9 minimal medium. For affinity purification of GST fusion proteins on glutathione-agarose (Sigma), cells were washed and lysed in phosphate-buffered saline. MBP fusion proteins were purified in 40 mM Tris/HCl pH 8.0, 100 mM NaCl on amylose resin (New England Biolabs, Frankfurt am Main, Germany). GST and MBP fusion proteins were eluted with 20 mM glutathione, 0.1 M Tris/HCl pH 8.0 and 20 mM maltose, 40 mM Tris/HCl pH 8.0, 100 mM NaCl, respectively. For pull-down assays, 4 μg of GST or GST fusion proteins were incubated with 5 μg of MBP fusion proteins in pull-down buffer (150 mM NaCl, 20 mM imidazole pH 8.0, 2 mM MgCl_2_, 300 mM sucrose, 0.25% (v/v) Triton X-100) or (150 mM NaCl, 20 mM HEPES pH 8.0, 2 mM MgCl_2_, 300 mM sucrose, 0.25% (v/v) Triton X-100) for 20 min at 4 °C. Assays were performed as described before [55].

### Proximity ligation assays

For PLAs [19], MCF-7 shNit1 clone 6 and scrambled clone 4 were used. Cells (0.5×10^6^) per six-well plate were seeded on a coverslide. After 48 h, PLA (Duolink Assay, Olink Bioscience distributed by Sigma) was performed with monoclonal anti-Nit1 (1C3) and polyclonal anti-β-catenin (M14M) according to the manufacturer’s recommendations. Images were taken with an inverse fluorescence microscope (ApoTome, Zeiss, Jena, Germany) and quantified with Fiji Open Source software [56].

### *Xenopus* double axis formation

Capped XWnt1 mRNA and hNit1 mRNA was synthesized from linearized plasmid template using the mMESSAGE mMACHINE kit (Ambion, Life Technologies, Darmstadt, Germany). Four nanoliters of XWnt1 mRNA (5 pg) alone or in combination with hNit1 (500 pg) mRNA were injected into the equatorial regions of the two prospective ventral blastomeres of four-cell stage *Xenopus* embryos. The embryos were then incubated at 19 °C and axis duplication was scored after 36 h. For luciferase experiments, 100 pg of the *siamois*-*luciferase* construct [21] were co-injected with 5 pg Xwnt8 mRNA and/or 500 pg hNit mRNA into all four blastomeres of four-cell stage embryos and analysed at the gastrula stage 10.5.

### Generation of transgenic flies/larvae

Transgenic fly lines expressing dNitFhit or its sub-fragments dNit and dFhit under the control of *UAS* promoter were generated using the ΦC31 integration system developed previously in our lab [57]. Constructs based on pUAST-attB-FLAG vector backbone were injected into the *ZP-attP-86Fb* fly line harbouring a landing site on the third chromosome. To express the UAS-driven constructs the fly lines were crossed to *yw; engrailed-Gal4,UAS-CD8-GFP* line. *Engrailed-Gal4* drives the expression in posterior part of wing imaginal discs as marked by co-expressed green fluorescent protein. Third instar larva wing imaginal discs were isolated, fixed and stained by standard techniques using guinea pig anti-Senseless antibody (provided by Hugo Bellen). Pictures were captured on a Zeiss LSM710 confocal microscope.

### Chromatin immunoprecipitation

The chromatin immunoprecipitation analysis was performed as described in Weiske and Huber [58] using 3 μg of the polyclonal anti-Nit1 antibody and oligonucleotides given in Supplementary Table S1.
