# Supplementary material for: A novel role for the tumour suppressor Nitrilase1 modulating the Wnt/β-catenin signalling pathway
Source: Cell Discov. 2016 Jan 5;2:15039–. doi: 10.1038/celldisc.2015.39 (PMC4860965; doi:10.1038/celldisc.2015.39)
Supplement: Supplementary Tables S1 and S2 [file celldisc201539-s1.pdf]

Supplementary Table 1. Primers used for plasmid generation

Gene	Primers	
hNit1 NM_005600.2	forward	5'-GCGGAATTCAGATCTGCCACCATGCTGGGCTTCATCACCAGG-3'
	reverse	5'-GCGCTCGAGAGATCTAGACAGTGGGTGACCCAG-3'
mutagenesis of endogenous hNit1 BamHI site	forward	5'-CTTACCTATCCTTCAGCTTTTGG**C**TCCATTACAGGCCCAGCCC-3'
	reverse	5'-GGGCTGGGCCTGTAATGGA**G**CCAAAAGCTGAAGGATAGGTAAG-3'
hNit1 NM_001185094	forward	5'-GCGGGATCCGCCGCCATGGCTATCTCCTCTTCC-3'
hNit1+stop	reverse	5'-GCGGGATCCTTAAGACAGTGGGTGACCCAG-3'
hNit1-stop	reverse	5'-GCGGGATCCAGACAGTGGGTGACCCAG-3'
hNit1ΔC+stop	reverse	5'-GCGGGATCCTTACACAGGCAGGTGTCG-3'
hNit1ΔC-stop	reverse	5'-GCGGGATCCCACAGGCAGGTGTCGGCG-3'
hNit1C203A mutagenesis	forward	5'-GGCAAGATTGGTCTAGCTGTCGC**C**TATGACATGCGGTTCCCTGAA-3'
hNit1C203A mutagenesis	reverse	5'-GTTCAGGGAACCGCATGTCATA**G**GCGACAGCTAGACCAATCTTGC-3'
hNit1-Drosophila linker-hFhit	reverse	5'-GCTGAACGACTGGGTTGGTTCCTTGCTGCGCAGTGGGTGACCCAGATTGCC-3'
	forward	5'-CGCAGCAAGGAACCAACCCAGTCGTTCAGCTTTGGCCAACATCTCATCAAG-3'
dNitFhit	forward	5'-CGCGGATCCGCCGCCATGTCAACTCTAGTTAATACC-3'
dNitFhit+stop	reverse	5'-CGCGGATCCCTAGCTTATGTCCGTCAG-3'
dNitFhit-stop	reverse	5'-CGCGGATCCGCTTATGTCCGTCAGAAA-3'
dNit1-stop	reverse	5'-GCGGGATCCTTAACGAAGGTTGTAGGCTGTTAAG-3'
dFhit	forward	5'- GCGGGATCCGCCGCCATGACCCAGGATCGACCATTTG-3'
hTCF-4	forward	5'-CGCGAATTCCATGCCGCAGCTGAACGGC-3'
□ΔNTCF-4	forward	5'-CGCGAATTCCGAAAACTCCTCGGCA-3'

Supplementary Table 2. Primers and UPL probes for qPCR

Gene	Primers		FAM dye-labeled Probe
human Nit1	forward	5´-CTGCGAATGGTTTTGGCTA-3´	#9
	reverse	5´-GGGACAGGAATCTGTGAGGA-3´	
human sp5	forward	5´-GGGGAGACTCAGCAGACG-3´	#36
	reverse	5´-TGGGTCCCTATGTCCGAAG-3´	
human GAPDH	forward	5´-AGCCACATCGCTCAGACAC-3´	#60
	reverse	5´-GCCCAATACGACCAAATCC-3´	

Details on the Universal Probe Library technology can be found at http://www.roche-applied-science.com/shop/CategoryDisplay?catalogId=10001&tab=&identifier=Universal+Probe+Library#tab-0
