# Supplementary material for: A novel role for the tumour suppressor Nitrilase1 modulating the Wnt/β-catenin signalling pathway
Source: Cell Discov. 2016 Jan 5;2:15039–. doi: 10.1038/celldisc.2015.39 (PMC4860965; doi:10.1038/celldisc.2015.39)
Supplement: Supplementary Figure S1–S8 [file celldisc201539-s2.pdf]

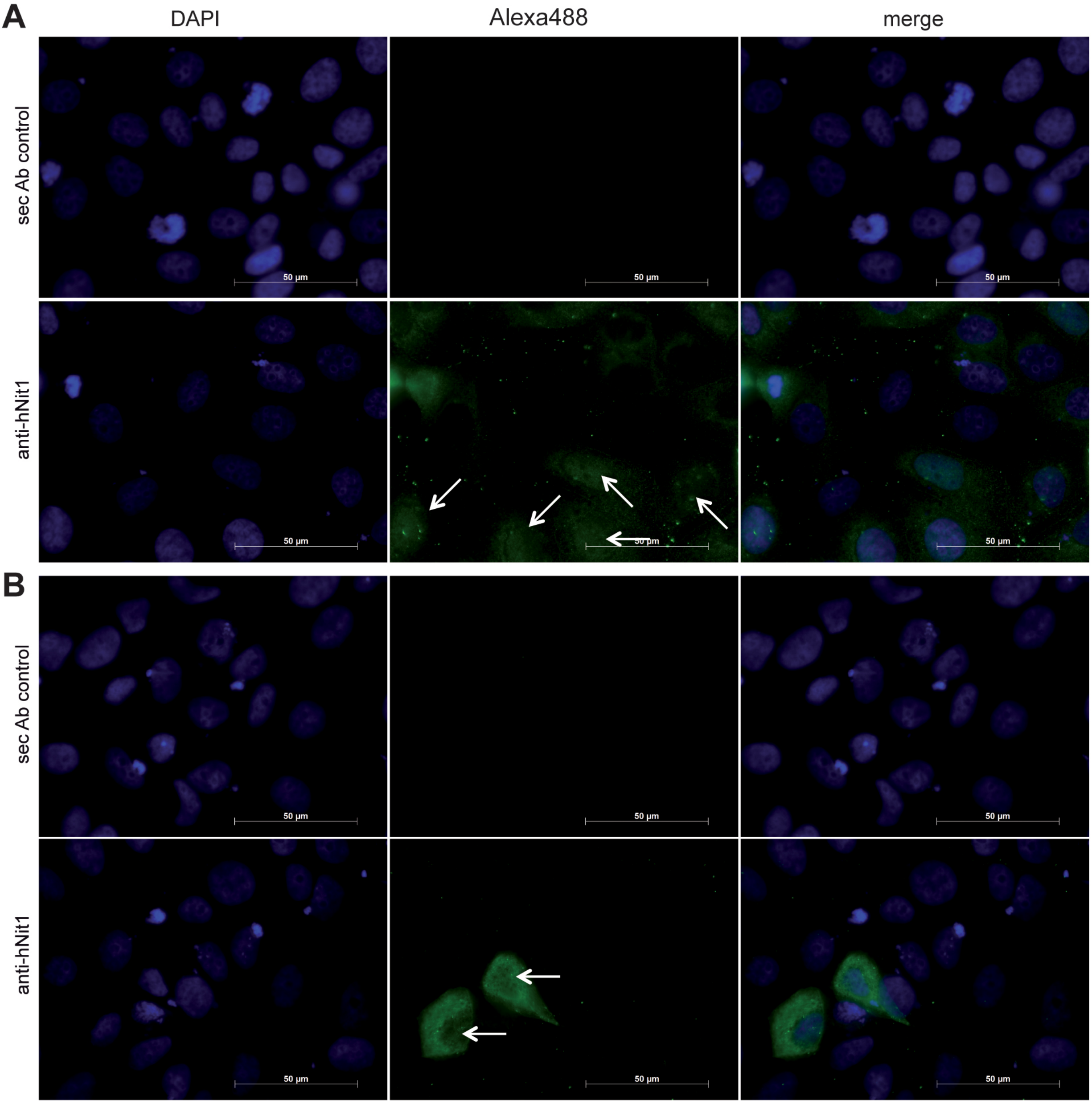

Supplementary Figure S1: A) MCF-7 cells were fixed in 3% formaldehyde and permeabilized with 0.5% (v/v) Triton X-100 and stained with anti-Nit1 antibody. Secondary antibody alone was used as a control. Cells with nuclear staining are marked with an arrow. B) MCF-7 cells were transiently transfected with hNit1 and stained as above. Exposure time was reduced compared to the images in A to specifically detect the overexpressed hNit1 which predominatly localizes to the cytosol but also in the nuclei. The bar represents 50 μm.

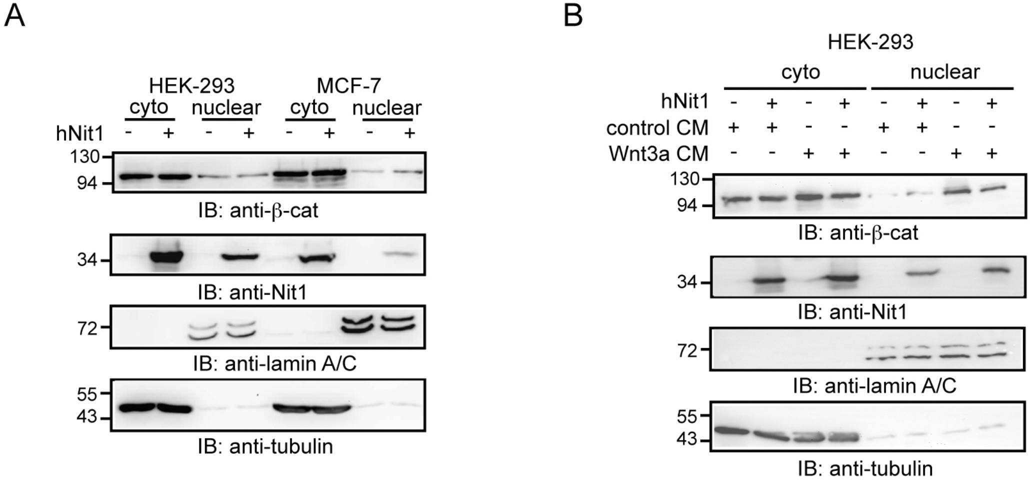

Supplementary Figure S2: A) β-Catenin distribution between cytosolic and nuclear fraction is not changed by overexpression of hNit in HEK-293 and MCF-7 cells. B) Stimulation of HEK-293 cells with Wnt3a-conditioned medium results in an increase of cytosolic and nuclear β-catenin. Transient transfection of hNit1 results in a reduced amount of nuclear β-catenin.

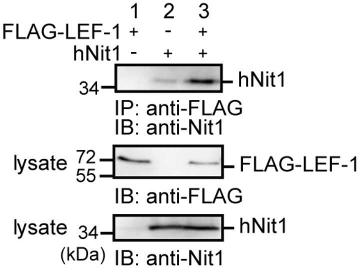

Supplementary Figure S3: Co-immunoprecipitation of hNit1with FLAG-LEF-1 from lysates of transiently transfected HEK-293 cells.

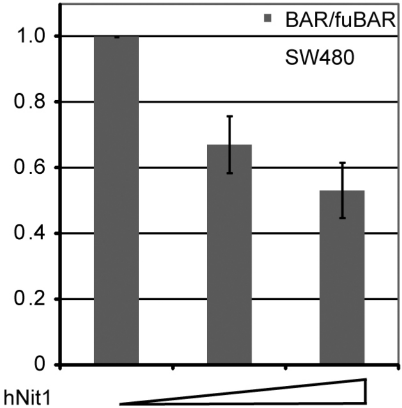

Supplementary Figure S4: Expression of hNit1 in SW480 colon carcinoma cells carrying an APC mutation resulting in constitutive active Wnt signalling induces a dose-dependent reduction of β-catenin transcriptional activity in BAR/fuBAR reporter gene assays (n=4).

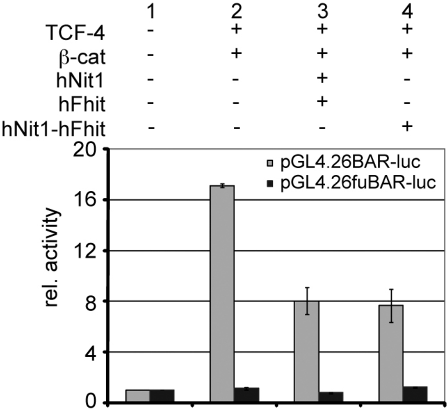

Supplementary Figure S5: An artificial hNit1-hFhit fusion protein exhibits similar repressive activity as co-transfection of the individual proteins in BARluc reporter gene assays in HEK-293 cells.

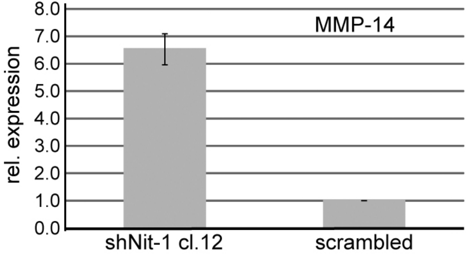

Supplementary Figure S6: Expression of the Wnt-target gene MMP-14 is upregulated in hNit1 knock-down MCF-7 cells as analysed by qRT-PCR (n= 8) and normalised to the scrambled clone.

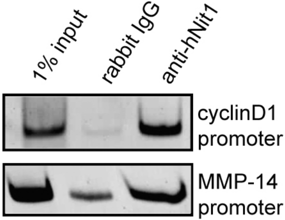

Supplementary Figure S7: HNit1 is recruited to the cyclin D1 and MMP-14 promoter. ChIP using anti-Nit-1antibody was performed with HEK-293 cells. Rabbit IgG was used as a control.

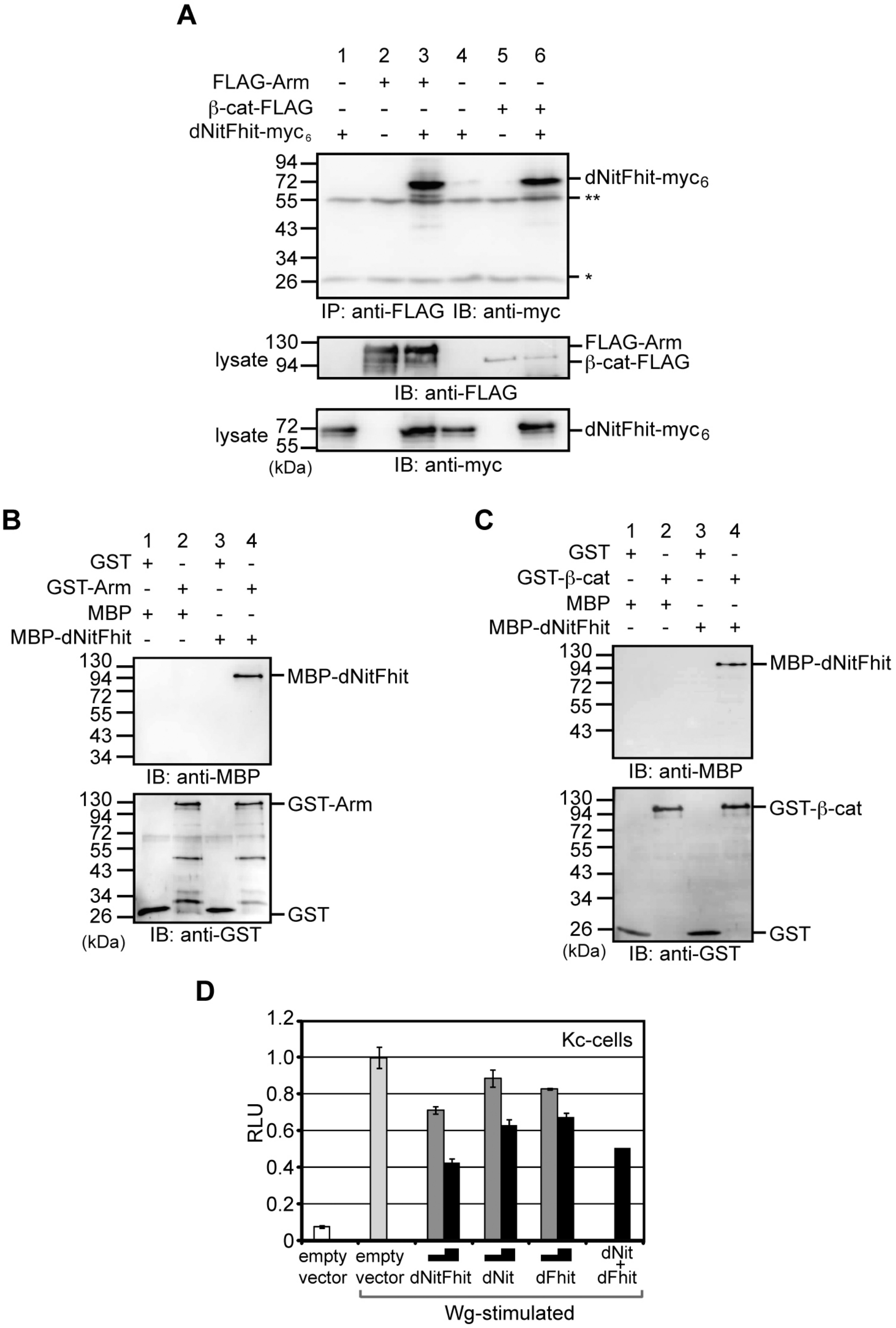

Supplementary Figure S8: DNitFhit binds to Armadillo and β-catenin and represses Wg-mediated transcription. (A) HEK-293 cells were transiently transfected with the indicated plasmids alone or in combination. Co-immunoprecipitation was performed with anti-FLAG M2 antibody and association of dNitFhit-myc6 was analyzed by Western blotting. Lysate controls are shown. *light chain, **heavy chain of the precipitating antibody. (B and C) Armadillo and β-catenin directly interact with dNitFhit. Purified recombinant fusion proteins were incubated as indicated and protein complexes were pulled down with GSH-agarose beads. Precipitated protein complexes were analysed by Western blotting with anti-MBP and anti-GST antibodies. Representatives of at least 3 independent experiments are presented. (D) Dose-dependent repression of wingful-luciferase reporter gene activity. Drosophila Kc cells were transfected with two concentrations (110 ng: grey bars, or 220 ng: black bars) of expression vector encoding dNitFhit or its sub-fragments dNit and dFhit under the control of actin5 promoter as indicated. Cells were stimulated with Wg-conditioned or control medium 48 h after transfection for additional 24 h and luciferase activities were then measured in triplicate and normalised to Renilla-luc activity. The levels of Wg-stimulated cells parallel transfected by empty vector were set as 1 (100%), error bars represents standard deviations. Representative results are shown.
